# Gems of Pen Arts

**Published:** 1871-12

**Authors:** 


*Gems of Pen Art.* By Charles B. Knowlton.

This beautiful little work, filled with truly fine gems in the art of writing, has been handed us by the author. Although not a medical work we cannot forego the pleasure of giving it a notice in our pages. The work comprises over thirty pages of matter pertaining to the art of writing, some dozen of which are taken up with instructions to the learner, the balance of the work being devoted to specimens of the different styles of writing and to some very beautiful pen drawings.

The engraving is done on fine thick paper and presents a fine appearance. The theoretical part comprises some valuable hints, and cannot fail to form a valuable adjunct to the school and counting-room.

